# Nodular Lymphocyte‐Predominant Hodgkin Lymphoma: Update on Biology and Treatment

**DOI:** 10.1002/hon.70080

**Published:** 2025-06-15

**Authors:** Peter Borchmann, Dennis A. Eichenauer, Gerard Frigola, Elias Campo

**Affiliations:** ^1^ First Department of Internal Medicine Center for Integrated Oncology Aachen Bonn Cologne Dusseldorf University of Cologne Cologne Germany; ^2^ German Hodgkin Study Group (GHSG) University Hospital Cologne Cologne Germany; ^3^ Laboratory of Pathology Hospital Clinic Barcelona University of Barcelona Barcelona Spain; ^4^ Institute for Biomedical Research August Pi i Sunyer (IDIBAPS) Barcelona Spain

**Keywords:** Hodgkin lymphoma, immuno‐architectural patterns, lymphoma transformation, nodular lymphocyte‐predominant B‐cell/Hodgkin lymphoma, T‐cell and histiocyte reach large B‐cell lymphoma

## Abstract

Nodular lymphocyte‐predominant B‐cell/Hodgkin lymphoma (NLPB/HL) accounts for roughly 5% of all Hodgkin lymphoma (HL) cases. In contrast to classic HL (cHL), the malignant cells in NLPB/HL lack CD30 expression but maintain a complete phenotype of mature B‐cells including CD20 and IgG, but occasionally show IgD expression. The tumor cells are known as lymphocyte‐predominant (LP) cells and grow in a T‐cell and histiocyte rich microenvironment against a background of follicular dendritic cells. Transformation to large B cell lymphoma may occur and requires excisional lymph node biopsies. Most patients with NLPB/HL present in early stages. The disease usually has an indolent clinical course. Approaches applied in cHL result in very good outcomes with only limited excess mortality in comparison with the general population. Activity has also been demonstrated for rituximab‐containing regimens commonly used in indolent B‐cell non‐Hodgkin lymphoma. The present article reviews pathological characteristics, treatment options and tools that might improve risk stratification in NLPB/HL.

## 1 Introduction

Nodular lymphocyte‐predominant Hodgkin lymphoma (NLPB/HL) is a rare entity that was initially classified as a subtype of Hodgkin lymphoma, but its biological, pathological and clinical features are different from classic Hodgkin lymphomas (cHL). Based on these differences and the close relationship of this entity with the T‐cell histiocyte rich large B cell lymphoma (THRLBCL), the 2022 International Consensus Classification of lymphoid neoplasms (ICC) proposed a change of the name to nodular lymphocyte predominant B‐cell lymphoma (NLPBL) [1, 2]. The fifth edition of the WHO classification retains the term of NLPHL. This condition typically affects the cervical, axillary, and inguinal lymph nodes of young men aged 30–50 years. Involvement of mediastinal or abdominal lymph nodes is very rare. Most patients present with early‐stage disease and generally have an indolent course, but a minority of patients may have a more aggressive behavior and transformation to large B cell lymphoma. In this review, we will address the key pathological and biological characteristics of these tumors with their clinical implications.

## 2 Pathology

Histologically, nodular lymphocyte predominant B‐cell/Hodgkin lymphoma (NLPB/HL) is characterized by a proliferation of scattered large tumor cells growing against a background of follicular dendritic cells with abundant reactive small B lymphocytes. The tumor cells are known as lymphocyte‐predominant (LP) cells and have folded nuclei and inconspicuous nucleoli. Immunophenotypically, LP cells are strongly positive for mature B‐cell markers CD20 and CD79a, as well as B‐cell transcription factors PAX5, OCT2, and BOB1, suggesting a preserved B‐cell transcription program. LP cells express EMA and exhibit a late germinal center B‐cell phenotype (MEF2B and BCL6 positivity, weak or absent CD10 and MUM1 expression). These cells are often surrounded by T follicular helper cells (TFH) cells forming rosettes, highlighted by PD1 [3], forming an immunological synapse [4]. Unlike Reed‐Sternberg cells in cHL, CD30 is usually negative but it may be seen in scattered immunoblasts outside the nodules. CD15 is also negative but may be expressed in rare cases [5]. Epstein‐Barr virus is negative, although rare positive cases have been reported, which have to be interpreted with caution [6, 7]. LP cells have clonal rearrangements of the immunoglobulin heavy chain gene with somatic hypermutations and ongoing mutations, suggesting the presence of functional B‐cell receptors and continued antigen exposure [8, 9, 10]. LP cells express mainly IgG, with a subset of IgD and light chain restriction [11]. The hypothesis of B‐cell receptor chronic antigenic stimulation has recently been strengthened with the analysis of B‐cell evolutionary trajectories in cases of relapsed or transformed NLPB/HL by NGS sequencing of immunoglobulin heavy chains [12], and by the identification of B‐cell receptor reactivity against *Moraxella catarrhalis* in a subset of IgD + NLPBL cases, and against *Rothia mucilaginosa* in other cases [13, 14].

## 3 Immuno‐Architectural Patterns

Different histological patterns have been described depending on the location of the LP cells and the composition of the microenvironment [15]. The majority of cases show a nodular growth pattern characterized by abundant reactive small B lymphocytes, some epithelioid histiocytes and scattered LP cells. Depending on the shape of the nodules, nodular or serpiginous, these cases are subdivided into patterns A and B. In both patterns the LP cells are within the reactive nodules, whereas in pattern C, numerous LP cells are outside of them. Pattern D still has a nodular architecture, although the nodules contain more T‐cells rather than B‐cells. Both patterns E and F are diffuse. Pattern E resemble a THRLBCL transformation, but the presence of residual nodules with follicular dendritic cell meshwork and T‐cell rosettes can help differentiate this pattern from THRLBCL. Pattern F displays numerous B‐cells with scattered LP cells interspersed with T‐cell aggregates in a moth‐eaten appearance.

These histopathological patterns differ in its clinical presentation and have been related with prognosis. Patterns A/B are usually associated with early/intermediate clinical stage, pattern C with an intermediate stage and patterns D/E with advance disease. Patterns C, D, and E have been associated with more aggressive clinical behavior, higher overall relapse rate, and increased rate of early relapses [16, 17, 18, 19, 20, 21, 22, 23]. A recent study has shown that pattern E was the only one significantly associated with worse progression free survival, overall survival and increased rate to large cell transformation [23]. Despite these associations, architectural patterns are not yet used to guide treatment in NLPB/HL, and the overall prognosis depends largely on the clinical stage [20, 21, 22, 23, 24], but recognizing them is crucial for differentiating NLPB/HL from histological similar conditions and to provide information in future prospective studies [23]. To avoid underrepresentation from core biopsies, excisional biopsy of a lymph node is recommended in these cases [1, 25].

The microenvironment of NLPB/HL shows a continuum from patterns A to E and THRLBCL, with decrease of CD4 T cell and increasing expansions of GZB+ natural killer and CD8+ T‐cells, PD1+ CD8+ T‐cells, and CD163+ macrophages, including a PD‐L1+ subset [26, 27]. Despite a tolerogenic immune response signature identified in THRLBCL cases compared to NLPB/HL [28], both refractory NLPB/HL cases with pattern E and THRLBCL show increased CD8+ T‐cells and type M1 macrophages compared to responders [7].

## 4 Molecular and Genetic Features

Genetic studies comparing NLPB/HL patterns A/B, NLPB/HL pattern E, and THRLBCL have shown that, although THRLBCL have a greater number of genomic alterations than NLPB/HL, the three entities have common gains of 2p16.1 and losses of 2p11.2 and 9p11.2 [29]. Gene expression analysis show similar profiles in microdissected tumor cells from NLPB/HL and THRLBCL [30]. *BCL6* rearrangements have been observed in 50% of NLPB/HL. Mutations in *DUSP2*, *JUNB*, *SGK1, JAK2*, *STAT6*, and *SOCS1* have been recurrently described in NLPB/HL [31] and THRLBCL [32], also supporting the relationship between both entities.

## 5 Large B‐Cell Lymphoma Transformation

Large B‐cell transformation in NLPB/HL has been recognized in 8.5% of tumors with pattern E whereas only in 1.7% of other histological patterns [23]. It seems to occur mainly in older patients and IgD‐negative tumors. Most studies suggest that DLBCL transformed from NLPB/HL tend to have a better prognosis than de novo DLBCL [33, 34, 35]. Histologic transformation may be present at diagnosis or occur many years after. Three histological patterns of the DLBCL component have been described: (1) large nodules of densely packed blasts (DLBCL transformation), (2) sharply demarcated blastic infiltrates with a T‐cell‐ and histiocyte‐rich microenvironment, where blasts comprise > 10% of the infiltrate (THRLBCL‐like transformation), and (3) cases with abundant necrosis and predominantly perivascular blastic infiltrates [36]. DLBCL cases with a nodular pattern and immunohistochemical positivity of the blasts for EMA could suggest a transformation from a NLPB/HL in apparently de novo DLBCL cases [36]. The mutational profile of DLBCL transformed from NLPB/HL shows alterations in genes of the NF‐kB (*SGK1*, *CARD11*, *JUNB*) and chromatin modification pathways, as well as frequent mutations in genes like *TET2*, *JUNB*, and *NOTCH2*, which are rare in de novo DLBCLs [37], and resembles the mutations of the ST2 subgroup of the LymphGen DLBCL genomic classification [38], Harvard's cluster 4 [39] and the TET2/SGK1 subgroup of the modified HMRN classification [40].

## 6 First‐Line Treatment

The first‐line treatment of NLPB/HL is based on the stage according to the Ann Arbor classification and the presence of risk factors.

Stage IA NLPB/HL without risk factors is usually treated with limited‐field radiotherapy (RT) alone. A large analysis included patients who had combined‐modality treatment (CMT) (*n* = 72), extended‐field RT alone (*n* = 49) or involved‐field RT alone (*n* = 108) within prospective German Hodgkin Study Group (GHSG) clinical trials. The 8‐year progression‐free survival (PFS) rates after CMT, extended‐field RT alone and involved‐field RT alone were ranging from 84.3% to 91.9%, the corresponding overall survival (OS) rates from 95.7% to 99.0% [41]. As alternative with a presumably reduced risk for the development of late toxicities, treatment with four weekly standard doses of rituximab was investigated in a phase II study comprising 28 patients. All patients responded to treatment [42]. However, the 10‐year PFS rate was only 51.1% and disease control thus worse than with RT alone [43].

NLPB/HL Although outcomes obtained with treatment strategies also used in cHL are very good, different institutions prefer to treat NLPB/HL with approaches commonly applied in indolent B‐cell non‐Hodgkin lymphoma (B‐NHL) [24, 44]. Data on the use of these approaches mostly derive from smaller retrospective analyses. A single‐center analysis included 27 NLPB/HL patients treated with R‐CHOP (rituximab, cyclophosphamide, doxorubicin, vincristine, prednisone) either alone or followed by consolidation RT. The 5‐year PFS was 88.5% [45]. A different analysis investigated 20 patients who had treatment with BR (bendamustine, rituximab). After a median follow‐up of 74 months, PFS rates at 36 and 68 months were 94% and 87%, respectively [46].

To summarize, limited‐field RT alone represents the standard approach for individuals with stage IA NLPB/HL without risk factors. The International Lymphoma Radiation Oncology Group recommends involved‐site RT alone for this patient group [47]. In early stages other than stage IA disease without risk factors, a brief ABVD‐based chemotherapy followed by limited‐field RT results in excellent PFS and OS outcomes and should therefore be considered as first‐line treatment.

In advanced NLPB/HL, very good disease control is achieved with interim PET‐guided escalated BEACOPP. However, such intensive therapy is likely not necessary in a relevant proportion of patients. Protocols usually given in indolent B‐NHL represent an alternative despite limited data not allowing firm conclusions regarding these regimens until now [22].

## 7 Treatment of Relapsed NLPB/HL

There is no standard approach for the management of NLPB/HL recurrence. In a relevant proportion of patients, single‐agent anti‐CD20 antibody treatment appears to be sufficient. Several prospective studies have indicated response rates close to 100% [48, 49, 50]. After a median observation time of 63 months, the median time to progression among 15 patients who had treatment with four weekly standard doses of rituximab within a phase II study was 33 months [48]. At 26 months, the 2‐year PFS estimate among 28 patients with relapsed NLPB/HL treated with eight weekly doses of ofatumumab was 80%. No deaths were reported [50]. Conventional chemotherapy optionally combined with rituximab and/or consolidation RT represents another option in relapsed NLPB/HL. This approach can especially be considered in patients who only had limited cumulative doses of chemotherapy at initial treatment. An analysis comprising 27 NLPB/HL patients treated with conventional chemotherapy at first relapse reported 5‐year PFS and OS rates of 68.0% and 77.8%, respectively [51]. Intensive second‐line treatment including high‐dose chemotherapy and autologous stem cell transplantation (ASCT) is only indicated in a minority of patients, that is those with poor‐risk characteristics such as disease recurrence < 24 months after the initial diagnosis, involvement of liver and/or bone marrow and disseminated disease at relapse after intensive first‐line treatment [51, 52]. An analysis using the database of the European Society for Blood and Marrow Transplantation included 60 patients with relapsed NLPB/HL who were treated with high‐dose chemotherapy and ASCT. Of these, more than 60% had presented with stage III‐IV disease at initial diagnosis; the time from initial diagnosis to high‐dose chemotherapy was < 24 months in 52% of cases. The 5‐year PFS and OS rates were 66% and 87% [53].

Treatment options in relapsed NLPB/HL thus range from single‐agent anti‐CD20 antibody treatment to high‐dose chemotherapy and ASCT. Salvage treatment is chosen individually based on factors such as time to relapse, previous treatment and disease burden at NLPB/HL recurrence [22].

## 8 Future Directions

As the overall prognosis of individuals with NLPB/HL is favorable, it is important to avoid overtreatment in low‐risk patients. At the same time, treatment intensity must be maintained in patients who are at an increased risk for treatment failure and death. A refined risk group allocation and the implementation of targeted drugs may result in an improved risk‐to‐benefit ratio of NLPB/HL treatment.

The lymphocyte‐predominant international prognostic score (LP‐IPS) includes the parameters age ≥ 45 years, stage III/IV disease, hemoglobin < 10.5 g/dL and splenic involvement. It allows the discrimination of risk groups with regard to PFS, OS, risk of histological transformation and lymphoma‐specific death [23]. Significant differences in terms of PFS and OS have also been demonstrated between patients with typical and variant histopathological growth patterns (GP) [17, 54]. However, the LP‐IPS and the GP have not been implemented into risk stratification systems yet. Novel approaches that might play a role in the future treatment of NLPB/HL include chimeric antigen receptor T‐cell therapy and bispecific antibodies. A study investigating the CD20xCD3 bispecific antibody mosunetuzumab in NLPB/HL (NCT05886036) is currently open for recruitment in North America [55].

## Disclosure

The authors have nothing to report.

## Conflicts of Interest

E.C. has been has received honoraria from Janssen, EUSA Pharma, Roche and ThermoFisher for speaking at educational activities and research funding from AstraZeneca and is an inventor on 2 patents filed by the National Institutes of Health, National Cancer Institute: “Methods for selecting and treating lymphoma types,” licensed to NanoString Technologies, and “Evaluation of mantle cell lymphoma and methods related thereof.” E.C. has licensed the use of the protected IgCaller algorithm for Diagnóstica Longwood and Sohia genomics.

## Peer Review

The peer review history for this article is available at https://www.webofscience.com/api/gateway/wos/peer-review/10.1002/hon.70080.

## Data Availability

The authors have nothing to report.
